# Correction: Suh Nchang et al. Knowledge about Asymptomatic Malaria and Acceptability of Using *Artemisia afra* Tea among Health Care Workers (HCWs) in Yaoundé, Cameroon: A Cross-Sectional Survey. *Int. J. Environ. Res. Public Health* 2023, *20*, 6309

**DOI:** 10.3390/ijerph21010080

**Published:** 2024-01-11

**Authors:** Abenwie Suh Nchang, Lahngong Methodius Shinyuy, Sandra Fankem Noukimi, Sylvia Njong, Sylvie Bambara, Edgar M. Kalimba, Joseph Kamga, Stephen Mbigha Ghogomu, Michel Frederich, Jean Lesort Louck Talom, Jacob Souopgui, Annie Robert

**Affiliations:** 1 Department of Epidemiology and Biostatistics (EPID), Institute de Recherche Expérimentale et Clinique (IREC), Public Health School, Université Catholique de Louvain (UCLouvain), 1200 Brussels, Belgium; sylvie.njong@student.uclouvain.be (S.N.); sylvie.bambara@amphealth.org (S.B.); annie.robert@uclouvain.be (A.R.); 2 Laboratory of Pharmacognosy, Department of Pharmacy, Center of Interdisciplinary Research on Medicine (CIRM), University of Liege, 4000 Liège, Belgium; ms.lahngong@doct.uliege.be (L.M.S.); m.frederich@uliege.be (M.F.); 3 Embriology and Biotechnology Laboratory, IBMM-ULB, 6041 Gosselies, Belgium; sandra.fankem.noukimi@ulb.be (S.F.N.); jacob.souopgui@ulb.be (J.S.); 4 King Faisal Hospital, Kigali 2534, Rwanda; edgar.mk@kfhkigali.com; 5 Biotechnology Unit, University of Buea, Buea P.O. Box 63, Cameroon; josephkamga700@yahoo.com (J.K.); stephen.ghogomu@ubuea.cm (S.M.G.); 6 Aumônerie—Hôpital du Jura bernois SA, 2740 Moutier, Switzerland; jean.louck@hjbe.ch

**Error in Table**

In the original publication [1], there was a mistake in **Table 3** as published. **The error made was that Table 4 was inserted twice as Table 3 and Table 4, while Table 3 was omitted. Table 3 was supposed to present factors associated with knowledge about the existence and definition of asymptomatic malaria among HCWs. While Table 4 presents the factors associated with knowledge about diagnosis and treatment of asymptomatic malaria among HCWs**. The corrected **Table 3** appears below. The authors state that the scientific conclusions are unaffected. This correction was approved by the Academic Editor. The original publication has also been updated.

## Figures and Tables

**Table 3:** Factors associated with knowledge about the existence and definition of asymptomatic malaria among HCWs.

Sample Characteristic	Knowledge of Existence of ASM				Knowledge of Definition of ASM					
	*n*	%	OR (95% CI)	*p*-Value	AOR (95% CI)	*p*-Value	*n*	%	OR (95% CI)	*p*-Value
**Total**	100	88.0					88.0	83.0		
***Age of respondent (years)***				>0.99						0.23
18–29	25	88.0	1		NI		22	90.0	2.45 (0.51–11.84)	
30–65	75	88.0	1				66	80.3	1	
***Gender***				0.24		0.07				0.58
Female	80	90.0	1		1		72	81.9	1	
Male	20	80.0	0.44 (0.12–1.66)		0.16 (0.22–1.15)		16	87.5	1.54 (0.31–7.63)	
***Professional category***				0.01 *		0.03 *				0.11
Nursing assistant	26	69.2	0.17 (0.03–0.91)		0.07 (0.01–0.77)		18	72.2	0.10 (0.01–0.99)	
State registered nurse	31	96.8	2.37 (0.20–26.94)		1.66 (0.68–19.89)		30	80.0	0.16 (0.02–1.43)	
Degree nurse	15	93.3	1.08 (0.09–12.95)		0.57 (0.03–9.32)		14	78.6	0.15 (0.14–1.57)	
Medical doctor (Physician)	28	92.9	1		1		26	96.2	1	
***Longevity of service (years)***				0.27						0.36
0–5	49	89.8	0.68 (0.12–3.74)		NI		44	88.6	2.34 (0.64–8.62)	
6–10	23	78.6	0.41 (0.05–1.59)				18	77.8	1.05 (0.25–4.42)	
>10	28	92.1	1				26	76.9	1	
***Practicing site***				0.21		0.73				0.10
Rural Hospital	50	78.3	2.19 (0.61–7.80)		1.31 (0.29–5.91)		46	89.1	2.56 (0.80–8.25)	
Urban Hospital	50	71.4	1		1		42	76.2	1	

* Statistically significant *p*-value.
